# Smoking habits of Greek preschool children's parents

**DOI:** 10.1186/1471-2458-7-112

**Published:** 2007-06-14

**Authors:** Constantine I Vardavas, Dimitrios Athanasopoulos, Evaggelia Balomenaki, Dora Niaounaki, Manolis K Linardakis, Anthony G Kafatos

**Affiliations:** 1 Preventive Medicine and Nutrition Clinic, Department of Social Medicine, School of Medicine, University of Crete, PO Box 2208, Heraklion 71003, Crete, Greece; 2 Pediatrics Clinic, Chania General Hospital, Crete, Greece; 3 Chania Office of primary education, Crete, Greece

## Abstract

**Background:**

Smoking is Greece's largest public health threat. Greece has the highest adult smoking prevalence among all E.U countries, which in turn possibly predisposes Greek children and adolescents to smoke. The purpose of our study was to research into the smoking habits of preschool children's parents since children of that age could be vulnerable to parental negative role modeling and to investigate into the necessity of conducting a public health awareness programme aimed at the general population.

**Methods:**

A cross-sectional study was performed on the parents of children enrolled in kindergarten in western Crete-Greece (2809 parents), and interviewed during the 2004–2005 Cretan school health promotion programme.

**Results:**

63% of households had at least one parent a current smoker and in 26% both parents were found to be current smokers. Smoking prevalence among adults with preschool children was estimated at 44% (52% of fathers and 36% of mothers). Paternal education and nationality were statistically significantly related to smoking (p < 0.001), unlike place of residence (p = 0.862) and level of maternal education (p = 0.132).

**Conclusion:**

Smoking prevalence is high even among parents with preschool children. Taking into account the parents' significant primary role in the children's upbringing and the effect that parental induced passive smoking has on children's health and health attitude; one can deduce that the health of Greek children is under threat. It is of major importance that educational and policy intervention measures are implemented to reduce such a situation that could contribute to promoting the initiation of smoking among Greek adolescents.

## Background

Smoking is a major public health threat, and has been characterized as the cause of the largest number of preventable deaths worldwide [1]. Greece is among the countries that face a serious smoking problem [2,3]. It has been estimated that Greece has the highest adult smoking prevalence among all countries in the European Union (E.U.) with an estimated 40% of the adult population current daily smokers [4]. Adolescents also have a serious smoking problem. Smoking prevalence differs according to place of residence, with inner city 15 year-old adolescents found to have a higher smoking prevalence of 32% in contrast to the number of adolescent smokers in Crete where only 10% smoke daily [5,6]. According to the findings of the Greek cohort of the Global Youth Tobacco Survey among 13–15 year olds throughout Greece 32.2% had ever smoked and approximately 1 in 4 of ever smokers had initiated smoking before the age of 10. [7]

Parents can affect the health and lifestyle of their children through role modeling, either positively or negatively, by influencing their attitudes towards health and substance abuse. Parents, close friends and older siblings can potentially influence children to smoke. Exposure to their parents' smoking habits may lead adolescents to develop the perception that smoking is a normative adult behavior [8]. It has been found that paternal and maternal smoking statuses are strong predictors not only of child smoking onset but as well as transition from initiation to monthly and then to daily cigarette use [9,10].

Parents that smoke not only act as negative role models for their children but also, unless precautions are taken, expose them to environmental tobacco smoke (ETS). It has been shown in numerous studies that exposure to ETS during childhood may lead to serious health problems. Children, due to higher ventilation rates, inhale elevated levels of ETS for the same level of exposure and due to their smaller body mass are affected more seriously than adults. Exposure to ETS affects children's health in many ways, by predisposing them to cancer [11], cardiovascular disease [12], asthma and lower respiratory tract infections [13], neurological disorders [14], and has even been found to affect child cognitive abilities [15].

Since Greece has a high percentage of current smokers, children's familiarity to cigarettes due to parental smoking as well as ETS exposure in the household, could affect both their child's perception of smoking and their exposure to ETS. Since young children often imitate their parent's habits, the purpose of this cross-sectional study was to assess the smoking habits of parents with young preschool children and to investigate into the necessity of conducting a public health awareness programme aimed at the general adult population of Crete.

## Methods

### Field study

The Chania educational health intervention programme, is a six year educational course taught by school teachers to all primary school children in Chania, Crete and aimed at nutritional education, substance use prevention and physical activity promotion. The sample population of this study consisted of 2809 adults (1420 families) representing the parents of the 1755 children who had enrolled in the study. From a total of 121 preschools in the prefecture 118 participated in the educational study. Out of the remaining three, two did not operate that academic year and one was excluded due to its topographical inaccessibility and small number of children. Totally out of 2630 children listed in preschools, 77% (1988 children) accepted to participate and 1755 were finally examined at baseline along with the lifestyle habits of 2809 parents. Regarding the parental lifestyle habits 2809 adults were personally interviewed using a health habits questionnaire designed by our Preventive Medicine and Nutrition Clinic during their child's baseline health assessment at their children's preschool [16]. The characteristics of the adult population are shown in Table 1. The Greek Ministry of Education and the University of Crete's Ethics Research Council approved the study, while the governing body of the prefecture of Chania provided funding.

**Table 1:** Characteristics of the parental population

	**Father**	**Mother**
**N**	1398 ^a^	1411
**Age (years)**	38.8 ± 6.0 ^b^	33.8 ± 5.0
**Education**		
*University*	14.9 (201) ^c^	17.7 (239)
*Intermediate*	14.4 (194)	14.9 (201)
*High school*	55.3 (746)	59.5 (803)
*Primary or none*	15.4 (207)	7.9 (107)
**Residence**		
*Urban*	34.7 (493)	
*Semi Urban*	9.9 (141)	
*Rural*	55.4 (786)	
**Nationality**		
*Both Greek*	86.3 (1226)	
*One parent Greek*	5.7 (81)	
*Both non Greek*	8.0 (113)	

a. Although there where 1420 answered questionnaires, the differences between 1398 and 1411 were due to single parent families (17 in total)

b. The values are given as mean ± standard deviation [Student t test – equal variances not assumed] p < 0.001

c. The values are given as total % (N) [Chi-square test (χ^2^)] p < 0.001

### Statistical analysis

Descriptive measurements were used to define the characteristics of parents who participated in the study. The Chi-square test (χ^2^) was used to calculate the distribution of the parent population regarding parameters such as level of education, area of residence, nationality and level of smoking. The students' t test was used to check for differences between the age of the parents, the years they smoked and the number of cigarettes consumed per day. The statistical analysis was accomplished using SPSS 13.0.

## Results

Smoking prevalence among parents of preschool children in Crete was estimated at 44 %. A difference was noticed between paternal and maternal smoking status, with a total of 51.9 % of the adult male population current smokers in contrast to 36.1 % of adult females. Only 37.2 % of households have non-smoking parents, while 37.3 % and 25.5 % have one or both parents' smokers respectively. The average number of smoking years were found to differ according to gender, 18 ( ± 6) for males to 12 ( ± 5) in females, although one must take into account the mean difference in age between the two sexes, which is about 5 years. Males were also found to be heavier smokers averaging 23 ( ± 12) cigarettes/day compared to the 15 ( ± 9) cigarettes for females. Table 2 depicts the parental smoking status.

**Table 2:** Parental Smoking habits in brief

		**Father**	**Mother**	**p-value**
**Smoking Prevalence**				
*Current smoker*		51.9 (726)^1^	36.1 (510)	
*Non smoker*		30.8 (431)	52.2 (737)	< 0.001
*Ex smoker*		17.2 (241)	11.6 (164)	
**Years**				
*Current smoker*		18 ± 6 ^2^	12 ± 5	< 0.001
*Ex smoker*		14 ± 8	9 ± 6	< 0.001
**Quantity (cigarettes/day)**				
*Current smoker*		23 ± 12 ^2^	15 ± 9	< 0.001
*Ex smoker*		22 ± 15	12 ± 9	< 0.001
**Parental Smoking**				
*Both smokers*		25.5 (356) ^3^		
*One smoker*		37.3 (521)		
*Both non smokers*		37.2 (519)		

		**Father**		

		*Smoker*	*Ex/Non smoker*	**p-value**

**Mother**	*Smoker*	25.5 (356) ^4^	11.0 (153)	< 0.001
	*Ex/Non smoker*	26.4 (368)	37.2 (519)	

1. The values are given as % (N = individuals) [Chi-square test (χ^2^)]

2. The values are given as: mean ± standard deviation [Student t test – equal variances not assumed]

3. The values are given as % (N = families)

4. The values are given as total % (N = families) [Chi-square test (χ^2^)]

The analysis of selected variables, such as residence, nationality, paternal and maternal education is shown in Table 3 Place of residence did not seem to be significantly related to smoking status(*p *= 0.862), but nationality was (*p *< 0.001). Higher percentages of smokers were found in families with both parents of Greek nationality compared to those with only one or no parents of Greek background. The mother's level of education was not related strongly to maternal non-smoking (*p *= 0.132) unlike paternal level of education, which was strongly related to non-smoking status (*p *< 0.001).

**Table 3:** Percentage (and number) of selected variables of preschool children's parents in relation to their parental smoking status

	***Both smokers***	***One parent smoker***	***Both non-smokers***	**p-value**
	% (N^b^)			
**Residence**				
*Urban*	24.7 (120)	37.5 (182)	37.7 (183)	0.862
*Semi Urban*	29.3 (41)	42.9 (60)	27.9 (39)	
*Rural*	25.2 (195)	36.3 (279)	38.5 (296)	
**Nationality**				
*Both Greek*	27.1 (326)	36.7 (442)	36.2 (436)	0.001
*One parent Greek*	18.8 (15)	41.3 (33)	40.0 (32)	
*Both non Greek*	12.7 (14)	41.8 (46)	45.5 (50)	
**Paternal education**				
*University*	21.0 (42)	34.0 (68)	45.0 (90)	0.001
*Intermediate*	24.6 (47)	36.1 (69)	39.3 (75)	
*High school*	24.8 (183)	38.1 (281)	37.0 (273)	
*Primary or none*	33.8 (69)	35.8 (73)	30.4 (62)	
**Maternal education**				
*University*	20.5 (49)	39.3(94)	40.2 (96)	0.121
*Intermediate*	27.8 (55)	32.3 (64)	39.9 (79)	
*High school*	26.5 (209)	37.6 (297)	35.9 (283)	
*Primary or none*	27.4 (29)	34.0 (36)	38.7 (41)	

a. Chi-square test (χ^2^) (linear by linear association

b. Numbers in brackets refer to families, not individuals.

## Discussion

Regarding smoking prevalence, our results are similar to those found among adults in Athens with 51% of males and 39% of females classified as daily smokers [17]. The slight difference noted between females between Chania and Athens could either be attributed to the different study location between Crete and central Athens or due to the fact that our study only included young parents with children at preschool. It is possible that a certain number of mothers possibly quit smoking, so as to protect their children from harm. Although a number of studies correlate higher paternal and maternal educational status with non-smoking smoking [18-20], we did not find any such relation regarding maternal smoking status (*p *= 0.132). It is possible that our findings could be partly attributed to the fact that higher educated Greek women have higher smoking rates than those who are less educated [4]. An alarming aspect of our research was the fact that almost 70% of the male population and 50% of the female population are either current or ex-smokers. Taking into account the huge costs of smoking associated with personal health and the demands on health services, one can understand the economic and social burden carried by the Cretan population.

Only 37% of households in our sample were found to have parents that do not smoke. Therefore taking into account that the other 63% of households have at least one parent as a current smoker, one can deduce that it is possible that a large percent of Greek children could be exposed to ETS and the familiarity of cigarettes at home. Indeed it has been stated that there is a dose-response relationship between parental and adolescent smoking with the risk of a child smoking found to increase relatively to the number of parents that smoke [21]. Since young children are influenced by and learn from their parents' and siblings behavior, parents that smoke not only provide easy access to cigarettes but also act as a primary role model for the initiation to smoking and its acceptance. [22]. This newly learned behavior might not even arise until a long time later, during adolescence, where children may be posed to mimic their parents smoking behavior in order for them to feel more like adults [23]. On the other hand, if parents quit smoking, it has been found to reduce the likelihood of their children becoming smokers. [24]. In Greece, negative parental role modeling may work in synergy with other factors that effect adolescent smoking. Such factors are peer pressure at school, the ability to buy cigarettes (it is not illegal to sell tobacco products to minors in Greece), their low cost compared to other European Countries and dense tobacco advertising on billboards, in kiosks, on means of public transport and even on school bus stops.

Regarding ETS exposure we could not estimate the exact number of households that were smoke free. It is possible that exposure to ETS at home could be lower due to the existence of smoke-free household areas or we expect that the actual percentage of tobacco-smoke free households in our study to be even lower due to the fact that other members of the extended family who live or spend time in the house may also be smokers and expose the children involuntarily to ETS. This hypothesis has been proven by the fact that the preschool children whose parents participated in this study had elevated serum cotinine levels (a biomarker that quantifies exposure to ETS) with young boys but especially girls found to have elevated serum cotinine levels especially in households with both parents smokers (1.69 ng/ml) but also even in those households with both parents non-smokers (0.15 ng/ml) verifying our hypothesis that Greek preschool children are heavily exposed to ETS in their home. [25]

In this study, we were not able to ascertain whether or not people other than the parents smoke regularly inside the house, nor were we able to determine if the parents take some precautions to prevent their children from inhaling smoke. Further studies are needed to assess such parameters, to measure ETS exposure in the home and to quantify the exact percentage of houses that are smoke-free in Greece. The indications are that elevated adult and adolescent smoking rates in Greece could be partly attributable to parental role modeling, exacerbated by the fact that nicotine exposure of preschool children has been proven to predispose them to becoming adolescent smokers [26]. Conclusively, a substantial percent of preschool children in Crete grow up in an environment that holds a pro tobacco stance. Taking the above findings into account the adoption of a parental health promotion programme would be a positive step towards enducing parental smoking cessation and would also increase their awareness of the adverse health effects ETS has on their offspring.

## Competing interests

The author(s) declare that they have no competing interests

## Authors' contributions

CIV participated in the study design and drafted the manuscript. DA, EB and DN collected data, and helped with the projects' coordination. ML participated in the study design, performed the statistical analysis and helped to draft the manuscript. AK participated in its design and coordination and helped to draft the manuscript. All authors read and approved the final manuscript.

## Pre-publication history

The pre-publication history for this paper can be accessed here:


